# Discrepancies between Multi-Electrode LFP and CSD Phase-Patterns: A Forward Modeling Study

**DOI:** 10.3389/fncir.2016.00051

**Published:** 2016-07-15

**Authors:** Rikkert Hindriks, Xerxes D. Arsiwalla, Theofanis Panagiotaropoulos, Michel Besserve, Paul F. M. J. Verschure, Nikos K. Logothetis, Gustavo Deco

**Affiliations:** ^1^Computational Neuroscience Group, Department of Information, Center for Brain and Cognition Barcelona, Spain; ^2^Synthetic Perceptive Emotive and Cognitive Systems Lab, Center of Autonomous Systems and Neurorobotics, Universitat Pompeu Fabra Barcelona, Spain; ^3^Department Physiology of Cognitive Processes, Max Planck Institute for Biological Cybernetics Tubingen, Germany; ^4^Centre for Systems Neuroscience, University of Leicester Leicester, UK; ^5^King's College London, Institute of Psychiatry, Psychology and Neuroscience London, UK; ^6^Institucio Catalana de Recerca i Estudis Avancats (ICREA), Universitat Pompeu Fabra Barcelona, Spain

**Keywords:** volume conduction, local field potential (LFP), traveling wave, neural oscillations, forward modeling, current source density (CSD), phase-dynamics

## Abstract

Multi-electrode recordings of local field potentials (LFPs) provide the opportunity to investigate the spatiotemporal organization of neural activity on the scale of several millimeters. In particular, the phases of oscillatory LFPs allow studying the coordination of neural oscillations in time and space and to tie it to cognitive processing. Given the computational roles of LFP phases, it is important to know how they relate to the phases of the underlying current source densities (CSDs) that generate them. Although CSDs and LFPs are distinct physical quantities, they are often (implicitly) identified when interpreting experimental observations. That this identification is problematic is clear from the fact that LFP phases change when switching to different electrode montages, while the underlying CSD phases remain unchanged. In this study we use a volume-conductor model to characterize discrepancies between LFP and CSD phase-patterns, to identify the contributing factors, and to assess the effect of different electrode montages. Although we focus on cortical LFPs recorded with two-dimensional (Utah) arrays, our findings are also relevant for other electrode configurations. We found that the main factors that determine the discrepancy between CSD and LFP phase-patterns are the frequency of the neural oscillations and the extent to which the laminar CSD profile is balanced. Furthermore, the presence of laminar phase-differences in cortical oscillations, as commonly observed in experiments, precludes identifying LFP phases with those of the CSD oscillations at a given cortical depth. This observation potentially complicates the interpretation of spike-LFP coherence and spike-triggered LFP averages. With respect to reference strategies, we found that the average-reference montage leads to larger discrepancies between LFP and CSD phases as compared with the referential montage, while the Laplacian montage reduces these discrepancies. We therefore advice to conduct analysis of two-dimensional LFP recordings using the Laplacian montage.

## Introduction

Multi-electrode recordings of local field potentials (LFPs) offer the possibility to monitor cortical activity with high spatiotemporal resolution. A central finding in such recordings is that cortical LFPs, whether ongoing, induced, or evoked, are highly organized in space and time and exhibit complex propagation patterns. Propagating LFPs have been observed in multiple cortical regions, including primary and higher visual cortices (Sato et al., 2012; Zheng and Yao, 2012; Zanos et al., 2015), temporal and auditory cortices (Reimer et al., 2011; Townsend et al., 2015), primary and premotor cortices (Rubino et al., 2006; Takahashi et al., 2011), as well as in other structures, most notably the hippocampus (Lubenov and Siapas, 2009; Patel et al., 2013; Zhang and Jacobs, 2015). Thus, while LFPs have traditionally been studied almost exclusively in the temporal domain, the development of multi-electrode arrays has forced us to treat cortical activity as continuous in space. This implies that all functional roles of cortical oscillations and their temporal coordination (Fries, 2005) can be recast into a broader framework (Maris et al., 2016). Indeed, most putative roles of propagating waves are based on the idea that LFPs reflect neural excitability (Buzsáki et al., 2012; Einevoll et al., 2013; Reimann et al., 2013). Therefore, propagating waves could subserve any function that relies on spatiotemporal modulation of neural excitability, such as sensory attention and, more generally, prioritizing of information streams (Wu et al., 2008; Zanos et al., 2015), phase-encoding in case of oscillatory waves (Ermentrout and Kleinfeld, 2001; Agarwal et al., 2014), and stimulus integration and segregation (Wu et al., 2008; Reimer et al., 2011; Sato et al., 2012; Zheng and Yao, 2012).

To clarify the functional roles of propagating waves, their properties such as direction, speed, and amplitude are correlated with cognitive variables (Rubino et al., 2006; Zanos et al., 2015). Successfully linking cognition to neural dynamics therefore, depends on how closely LFP dynamics follow those of the underlying neural currents. Although LFPs are often identified with neural activity, they are generated by transmembrane currents that set up electric fields and concomitant currents in the surrounding tissue (Nicholson and Freeman, 1975; Buzsáki et al., 2012). Thus, the LFP recorded at any particular location reflects the integrated transmembrane currents from the surrounding tissue, a fact that complicates their interpretation. Although volume-conduction is what makes electrophysiological recordings possible, it also complicates their interpretation in terms of neural activity. Consider, as an example, a cortical rhythm that is organized as a delayed feedback loop between cortical layers. Since the LFP phase at a given cortical depth depends on the phases (and amplitudes) of the transmembrane currents in all cortical layers and the current oscillations exhibit phase-differences, the LFP phase does not correspond to that of the currents are any particular cortical layer. A similar ambiguity exists in interpreting the peaks and troughs of evoked potentials (Barth and Di, 1990). A recent study illustrates how identifying electric potentials with neural activity can have a substantial impact on cognitive theories (Hindriks et al., 2014).

The example treated in Hindriks et al. (2014) is concerned with the biophysical interpretation of alpha (7–13 Hz) oscillations as recorded with scalp EEG, which are known to propagate over the scalp with speeds in the range of 5–15 m/s and predominantly along the medial axis. This observation has lead to the hypothesis that the propagation of the underlying cortical currents is mediated by cortico-cortical axons, since the propagation velocities of action potentials in such axons fall within the same range. This *cortico-cortical hypothesis* has led to the idea that propagating alpha waves enable communication between frontal and occipital regions. Such communication would be enabled by the long wavelengths of EEG alpha oscillations, which, given their speeds, would be of the same order as the distance between frontal and occipital regions. This view assumes that the underlying cortical currents propagate with similar speeds. This assumption, however, is inconsistent with LFP recordings, which have shown alpha oscillations to propagate with velocities around 0.3 m/s and therefore suggest mediation by intra-cortical axons (which are known to conduct action potentials with velocities in the range 0.1–1 m/s). In Hindriks et al. (2014), this issue is solved by constructing an EEG volume-conductor model and using it to show that cortical currents propagating at 0.3 m/s can lead to speeds of EEG potentials in the observed range of 5–15 m/s. According to this *intra-cortical hypothesis*, alpha wavelengths are short (on the order of centimeters) which precludes a wavelength-based communication mechanism between frontal and occipital regions.

From a mesoscopic point of view, transmembrane currents are described in terms of a (volume) current source density (CSD) (Nicholson and Freeman, 1975; Mitsdorf, 1985). The CSD is a scalar function of location that describes how much current enters or leaves the extracellular medium per unit-of-volume. A positive/negative CSD signifies that current enters/leaves the extracellular medium and hence corresponds to hyperpolarization/depolarization of local neural populations. In CSD analysis of LFP recordings, regions with positive/negative CSD are commonly referred to as (current) sources/sinks (Mitsdorf, 1985). From a biophysical point of view, it is the CSD that corresponds to neural activity and hence is the main variable of interest. The aim of this study, therefore, is to assess how CSD and LFP phase-patterns are related and how different variables—in particular the inter-laminar organization of the CSD—change this relation. This is done by simulating three-dimensional CSDs, calculating the LFPs using a volume-conductor model, and by subsequently comparing their dynamics using appropriate indices. To calculate the LFP due to a given CSD we solve Poisson's equation (Nicholson and Freeman, 1975). In the context of electrophysiology, solving Poisson's equation is often referred to as *forward modeling* and the resulting model as a *volume-conductor model*. This biophysical approach has proven valuable in understanding the biophysical nature and physiological content of the LFP (Kajikawa and Schroeder, 2011; Linden et al., 2011; Buzsáki et al., 2012; Einevoll et al., 2013; Reimann et al., 2013).

In this study we simulate cortical LFPs as recorded with the Utah array, which is a two-dimensional array containing 100 electrodes, arranged in a 10 × 10 grid with 400 μ*m* inter-electrode spacing (Maynard et al., 1997), as such arrays are most frequently used to record two-dimensional cortical LFPs (Rubino et al., 2006; Takahashi et al., 2011; Zheng and Yao, 2012; Zanos et al., 2015). The aim of this study is to characterize the discrepancies between oscillatory CSD and LFP phase-dynamics and to identify the contributing factors such as the laminar organization and temporal frequency of the oscillations, and the electrode montage used. After describing the volume-conductor model (Section Volume Conductor Model), the CSD simulations (Section CSD Simulations), and how to compare LFP and CSD phase-dynamics (Section Comparison of LFP and CSD Phase-Patterns), we try to give the reader some intuition on how discrepancies between CSD and LFP phase-dynamics arise (Section The LFP as an Integrated Signal) and illustrate this with an analytical example (Section Phase-Contraction). In Sections LFP-CSD Phase-Coherence and LFP and CSD Propagation Speeds we consider volume-conduction effects in case of complex phase-patterns and traveling plane waves, respectively. In Section Laminar Contributions to LFP Oscillatory Phase we assess how phase-differences between neural oscillations in different cortical layers affect the LFP phase recorded at a given depth. Finally, in Sections The Average-Reference Montage, The Bipolar Montage, and The Laplacian Montage we assess the effects of switching to different electrode montages.

## Materials and methods

### Volume conductor model

LFPs are generated by transmembrane currents, which give rise to extra cellular electric fields and their corresponding electric potentials (Buzsáki et al., 2012). The transmembrane currents are commonly described on the mesoscopic level in terms of a (volume) *current source density C*, which has the dimension of current per unit-of-volume. *C* is a scalar function of cortical location and describes how much current leaves or enters the extracellular medium per unit-of-volume. The electric potential *V* (that is, the LFP) and the CSD are related through Poisson's equation:

(1) $$\begin{array}{l} \nabla •\sigma \nabla V=-C, \end{array}$$

where ∇ denotes the gradient operator, • the inner product, and σ denotes the conductivity tensor. We assume the tissue to be of infinite extent, since the head is much larger than the volume of interest. Furthermore, we assume the conductivity to be homogeneous and isotropic. Although this is a simplification, because the conductivity differs between tissue types (gray and white matter, cerebrospinal fluid, bone), and might be different in the intra- and inter-laminar directions (Logothetis et al., 2007), these assumptions been proven to give good results in modeling studies of LFPs (Riera et al., 2012; Reimann et al., 2013; Kajikawa and Schroeder, 2015) and will therefore serve as a good starting point. Under these assumptions, σ reduces to a scalar and the LFP is given by

(2) $$\begin{array}{l} V\left(x,y,z\right)=\frac{1}{4\pi \sigma }∭\frac{C\left(x^{\prime },y^{\prime },z^{\prime }\right)dx^{\prime }dy^{\prime }dz^{\prime }}{\sqrt{{\left(x-x^{\prime }\right)}^{2}+{\left(y-y^{\prime }\right)}^{2}+{\left(z-z^{\prime }\right)}^{2}}}, \end{array}$$

where we have used Cartesian coordinates (*x, y, z*), where (*x, y*) and *z* denote intra- and inter-laminar coordinates, respectively and where the integral is taken over the support of *C*. In particular, for a localized source of unit strength, located at the origin, that is *C*(*x, y, z*) = δ(*x, y, z*), the LFP is given by

(3) $$\begin{array}{l} V\left(x,y,z\right)=\frac{1}{4\pi \sigma \sqrt{x^{2}+y^{2}+z^{2}}}. \end{array}$$

Unlike in the case of EEG or ECoG, where the recording electrodes are located outside the tissue, the recording tips of intra-cortical electrodes can be arbitrarily close to the current sources. As Equation (3) shows, this leads to singularities when using localized sources to model the CSD. This can be dealt with in several ways (Pettersen et al., 2006; Riera et al., 2012; Reimann et al., 2013; Kajikawa and Schroeder, 2015). We avoid the singularity by assuming the CSD to be constant on small rectangular tissue volumes, in which case we can use the explicit formula derived in Hummer (1996).

**Figure 1:**
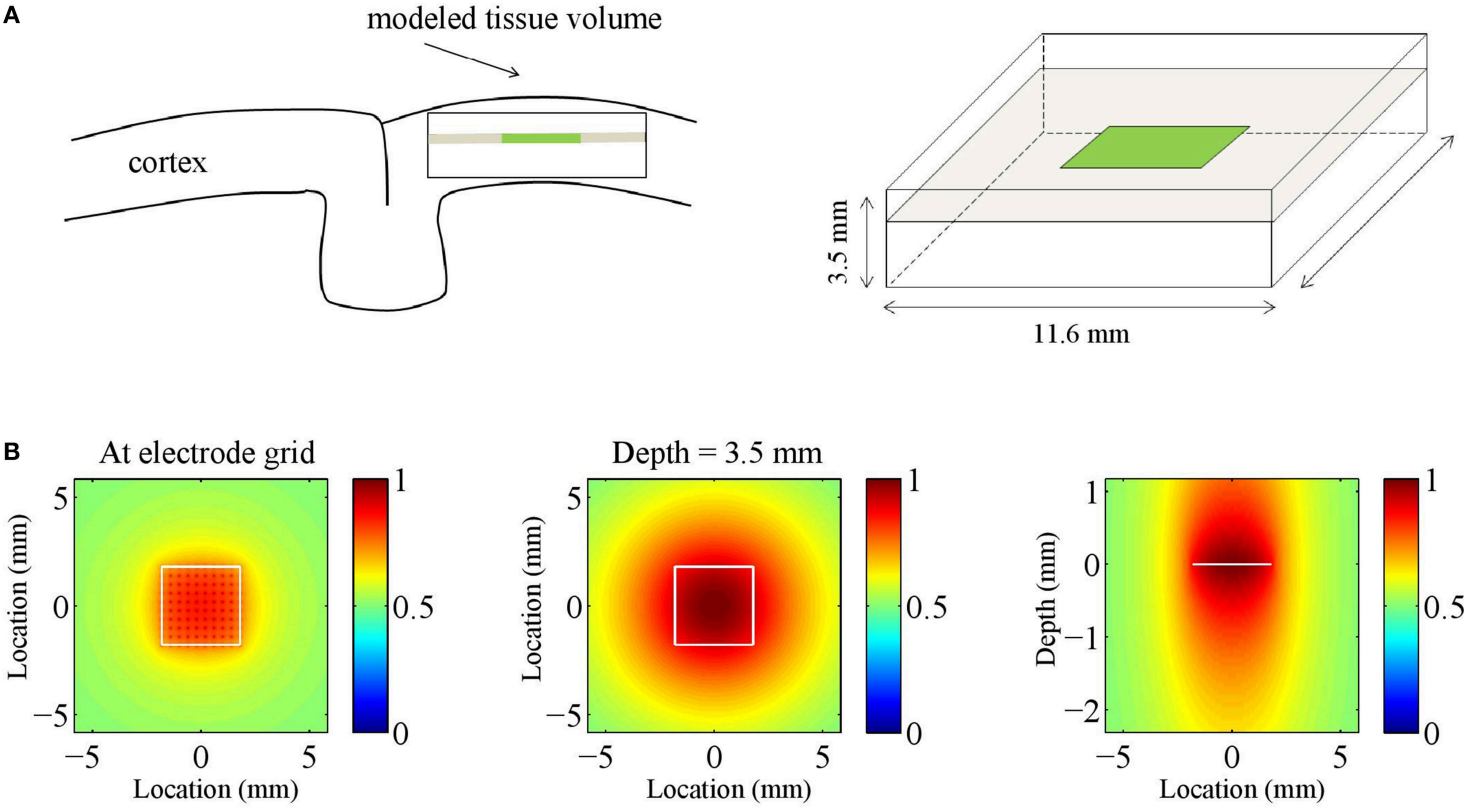
**Volume-conductor model. (A)** Schematic drawing of the modeled tissue volume (black square) and the placement of the electrode grid (green line). The dimensions of the volume are as indicated: an intra-laminar length of 11.6 mm and an inter-laminar length of 3.5 mm. The electrode grid is located at the horizontal center of the tissue volume at a depth of 1.15 mm. **(B)** The panels show the electrode-averaged leadfield sensitivity for three different cross-sections of the modeled tissue volume. Left and middle panel: intra-laminar cross-section at a depth of 1.15 mm (that is, at the height of the electrode grid) and 3.5 mm (that is, at the boundary between cortex and white-matter), respectively. Right panel: inter-laminar cross-section through the center of the electrode grid. The color-coded values are normalized. The white contours designate the boundary of the electrode grid.

We restricted the CSD to a rectangular tissue volume with intra- and inter-laminar lengths of 11.6 mm (equal to the length of the electrode grid (3.6 mm) plus a 4 mm extension along the side) and 3.5 mm, respectively. The inter-laminar length is roughly an upper bound for the thickness of primate cortex. The recording grid was arranged in a 10 × 10 electrode array with 400 μ*m* inter-electrode spacing and was placed at the intra-laminar center of the tissue volume, 1.15 mm deep. Figure 1A illustrates the arrangement.

By dividing the modeled volume into *K* cubes and assuming the CSD is constant on each cube, the relation between *C* and *V* can be written as follows:

(4) $$\begin{array}{l} V=GC, \end{array}$$

where *V* ∈ *R*^*N*×1^ denotes the vector containing the LFPs at the *N* = 100 electrodes and *C* ∈ *R*^*K*×1^ denotes the vector containing the CSD at the *K* source locations. The matrix *G* ∈ *R*^*N*×*K*^ is known as the (LFP) *leadfield matrix* (Grech et al., 2008) and its columns are known as *leadfields* and correspond to source locations: They describe how strong a source at that particular location contributes to the voltages recorded at the different electrodes. The volume was divided into *K* = 204^2^×61 = 2538576 cubes (204^2^ in each of the 61 intra-laminar planes) Figure 1B shows the electrode-averaged sensitivity of the leadfield for three different cross-sections through the tissue volume. The sensitivity was calculated by averaging *G* over rows (that is, over electrodes). The intra- and inter-laminar sensitivity of the LFP leadfield lead to volume-conducted contributions of neural activity from superficial and deep cortical layers, respectively, and form the physical basis for the non-local character of the LFP (Kajikawa and Schroeder, 2011).

### CSD simulations

We simulated neural activity inside the modeled tissue by specifying a complex-valued CSD *C*(*x, y, z, t*), where (*x, y, z*) denotes position in Euclidean coordinates, with *x* and *y* denoting the intra-laminar directions, *z* the inter-laminar direction, and *t* denotes time. The electrode grid is located at *z* = 0 and the positive *z*−axis points toward the pial surface. We focus on oscillatory LFPs and thus take *C* to be oscillatory, say with frequency *f*. Moreover, we assume the intra- and inter-laminar profiles of *C* to be independent, so that *C* can be decomposed as follows:

(5) $$\begin{array}{l} C\left(x,y,z,t\right)=C_{v}\left(z\right)C_{h}\left(x,y\right)C_{t}\left(t\right), \end{array}$$

where *C*_*v*_ and *C*_*h*_ denote, respectively, the inter- and intra-laminar CSD profiles (“ *h* ” stands for “horizontal” and “ *v* ” for “vertical”) and

(6) $$\begin{array}{l} C_{t}\left(t\right)=e^{i2\pi ft}, \end{array}$$

denotes its temporal profile.

The inter-laminar profile is modeled by a linear superposition of dipolar sink-source configurations. We will refer to these dipolar configurations as *generators* and model them by a linear superposition of two Gaussian profiles with amplitudes *A* and (1−ε)*A*, where 0 ≤ ε ≤ 1, opposite signs, inter-laminar locations *z*_0_+*L*∕2 and *z*_0_−*L*∕2 and common width σ_*v*_. Thus, in case of a single generator, the inter-laminar profile is given by

(7) $$\begin{array}{l} C_{v}\left(z\right)=Ae^{-{\left(z-\left(z_{0}+L∕2\right)\right)}^{2}∕2\sigma_{v}^{2}}-\left(1-\varepsilon \right)Ae^{-{\left(z-\left(z_{0}-L∕2\right)\right)}^{2}∕2\sigma_{v}^{2}}. \end{array}$$

**Figure 2:**
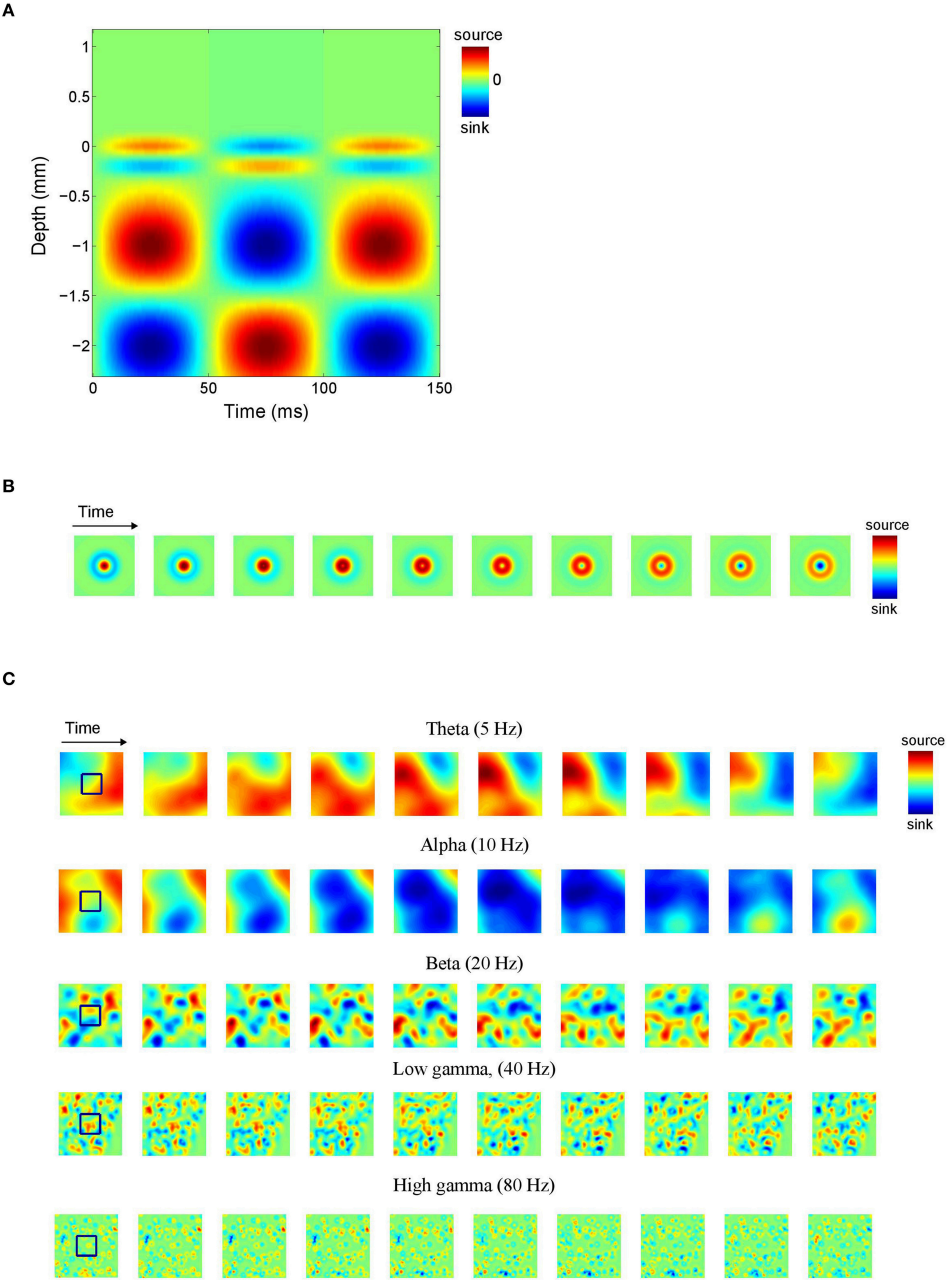
**Current source density (CSD) simulations. (A)** Simulated inter-laminar CSD component (color-coded) as a function of time and cortical depth (relative to the electrode grid). Red and blue correspond to current sources (CSD > 0) and sinks (CSD < 0), respectively. In this example the component was modeled by a superposition of two dipolar sink-source configurations (generators) located at depths of −0.1 and −1.6 mm and having lengths of 0.2 and 1 mm, respectively. The deep generator has twice the amplitude of the superficial generator and display in-phase 10 Hz oscillations. **(B)** Intra-laminar component of simulated isotropically propagating beta (20 Hz) oscillations originating from the center of the tissue volume. Initial phase was randomly chosen. **(C)** Intra-laminar component of simulated CSD constructed by superposing 100 isotropically propagating oscillations with random positions and initial phases. In **(B,C)** the timespan is half the oscillation period. The black squares denote the boundary of the electrode array. Parameters were set as in Table 1.

Note that the different signs lead to anti-phase oscillations of the two generator poles (the source and the sink), reflecting conservation of current in the extra cellular medium. For ε = 0, the poles of the generator are *balanced*, which is equivalent to the above mentioned current conservation. Neural sources, however, can be unbalanced or monopolar, due to ionic diffusion currents (Bédard and Destexhe, 2009; Riera et al., 2012). This is modeled by the parameter ε, where ε = 1 and ε = 0 correspond to balanced and monopolar sources, respectively, and values in between model unbalanced sources. We consider three values of ε: 0, 0.5, and 1, corresponding to balanced, unbalanced, and monopolar sources, respectively. Figure 2A shows an example of an inter-laminar CSD component (color-coded) as a function of cortical depth and time. It comprises two generators: a short superficial one (*L* = 0.2 mm and *z*_0_ = −0.1 mm) and a long deep one (*L* = 1 mm and *z*_0_ = −1.6 mm). The generators display 10 Hz oscillations. Other parameters were set as in Table 1.

**Table 1:** **CSD parameters, their symbols, and ranges/nominal values**.

**Parameter**	**Symbol**	**Range/Nominal value**
Oscillation frequency	*f*	5–80 Hz
Generator amplitude	*A*	1
Generator length	*L*	0.2–1 mm
Inter-laminar width (of generator poles)	^σ^ *v*	*L*∕3 mm
Propagation speed	*v*	0.1–0.2 m/s
Intra-laminar width (of isotropic waves)	^σ^ *h*	*v*∕3*f* mm
Level of current imbalance	ε	0, 0.5, 1

We used two models for the intra-laminar CSD profile *C*_*h*_. The first model is a superposition of isotropic waves. In case of a single wave, *C*_*h*_ is given by

(8) $$\begin{array}{l} C_{h}\left(x,y\right)=e^{i\varphi_{0}}e^{-i2\pi d\left(x,y\right)∕\lambda }e^{-d^{2}\left(x,y\right)∕2\sigma_{h}^{2}} \end{array}$$

where φ_0_ denotes the initial phase of the wave, σ_*h*_ its spatial width, *d*(*x, y*) denotes the intra-laminar distance of (*x, y*) to the center of the wave, and λ = *v*∕*f* denotes its wavelength, where *v* is the propagation speed. Figure 2B shows an isotropically propagating wave traveling away from the (intra-laminar) center of the tissue volume with a speed of *v* = 0.2 m/s and having a spatial width of σ_*h*_ = 1 mm. Figure 2C shows *C*_*h*_ modeled as a superposition of 100 such sources with random locations and initial phases for five different oscillation frequencies. Notice that with increasing frequency, the oscillations become less spatially coherent. The second model is a traveling plane wave, in which case *C*_*h*_ is given by

(9) $$\begin{array}{l} C_{h}\left(x,y\right)=e^{i\varphi_{0}}e^{i\left(k_{x}x+k_{y}y\right)}, \end{array}$$

where φ_0_ denotes the initial phase of the wave and *k*_*x*_ and *k*_*y*_ denote the wavenumbers in the *x*− and *y*−directions, respectively. The propagation speed of the wave is given by *v* = λ*f*, where $\lambda =1∕\sqrt{k_{x}^{2}+k_{y}^{2}}$ denotes the wavelength.

To cover the experimental LFP frequency range, we chose a representative value within the theta, alpha, beta, and (low and high) gamma frequency bands. Specifically, we set *f* to 5 Hz (theta), 10 Hz (alpha), 20 Hz (beta), 40 Hz (low gamma), and 80 Hz (high gamma). Generator amplitude *A* was set to the normalized value of 1 since it does not impact CSD and LFP phase-dynamics. In case of multipolar CSDs (that is, CSDs whose inter-laminar component comprises more than one generator) we do vary their relative amplitudes, however. The generator length *L* is identified with the space constant of passive dendrites, which takes values in the range 0.2–1 mm (Hindriks et al., 2014). In case of plane waves, the propagation speed was set to *v* = 0.2 m/s which is consistent with both LFP (Freeman et al., 2000; Rubino et al., 2006; Reimer et al., 2011; Takahashi et al., 2011; Zanos et al., 2015) and voltage sensitive dye (VSD) experiments (Slovin et al., 2002; Benucci et al., 2007; Wu et al., 2008; Sato et al., 2012). In the case of isotropic waves, *v* was set to a lower value (*v* = 0.1 m/s) because for superpositions of such waves, the effective propagation speed as estimated from the simulated data (see Section Comparison of LFP and CSD Phase-Patterns) is higher. We point out that the dynamics—and hence the effect of volume-conduction—of both the plane and isotropic waves effectively depends on the wavelength λ = *v*∕*f* so that a different value of *v* is equivalent to rescaling the frequency range. The intra-laminar width σ_*h*_ of isotropically propagating waves was set to σ_*h*_ = *v*∕3*f* (or equivalently, σ_*h*_ = λ∕3) which ensures that the wave is damped out within a distance of λ from its center, as frequently observed experimentally (Ermentrout and Kleinfeld, 2001). The inter-laminar width σ_*v*_ of the generator poles was set to *L*∕3 which ensures that the generator poles partially overlap. This choice reflects the fact that current sinks and sources are typically not separated by sourceless tissue, an observation that is probably due to the spatially continuous nature of the associated return currents that generate the LFP. The parameters and their ranges are listed in Table 1.

### Comparison of LFP and CSD phase-patterns

To characterize the phase-patterns of the simulated CSDs and LFPs we use two indices. In case of isotropically propagating waves we use the Kuramoto order parameter and in case of planar traveling waves, we use the (spatially-averaged) propagation speed. When applied to simulated CSDs, the CSDs are restricted to the locations of the grid electrodes to enable a direct comparison with the values obtained from the LFPs.

The *Kuramoto order parameter*, denoted by *r*, is defined by

(10) $$\begin{array}{l} r=|\langle e^{i\psi }\rangle |, \end{array}$$

where the brackets denote averaging over electrodes and the vertical bars denote taking the absolute value (Breakspear et al., 2010). The Kuramoto order parameter takes on values between 0 and 1 and quantifies the extent to which the phases ψ at different locations are aligned. We calculate this index both for the simulated LFPs as well as for the underlying CSDs and denote the values by *r*_*LFP*_ and *r*_*CSD*_, respectively.

The average propagation speed was calculated similarly as in Takahashi et al. (2011) and is based on the spatial gradient of the instantaneous phase-pattern ψ(*x, y*). In Cartesian coordinates, the spatial gradient is given by

(11) $$\begin{array}{l} \nabla \psi =\frac{\partial \psi }{\partial x}\hat{x}+\frac{\partial \psi }{\partial y}ŷ, \end{array}$$

where $\hat{x}$ and ŷ denote the standard two-dimensional Euclidean basis vectors. It is calculated by approximating the partial derivatives ∂ψ∕∂*x* and ∂ψ∕∂*y* by first-order finite differences.

Note that the spatial unwrapping of the phases used in calculating ψ can only be done accurately if δ < 2λ, where δ is the inter-electrode distance and λ is the wavelength of the oscillations. Since λ = *v*∕*f*, where *v* is the propagation speed and *f* the temporal frequency of the oscillations, this condition is equivalent to 2*v*∕*f* > δ. In this study, δ = 0.4 mm, *v*≥0.1 m/s, and *f* ≤ 80 Hz, so that this condition is satisfied.

The *average propagation speed*, denoted by $\overset{-}{v}$, is calculated as

(12) $$\begin{array}{l} \overset{-}{v}=\frac{2\pi f}{\langle \left||\nabla \psi |\right|\rangle }, \end{array}$$

where (as above) the brackets denote averaging over electrodes, *f* denotes the frequency of the oscillations, and the double vertical bars denote the Euclidean norm:

(13) $$\begin{array}{cc} {\left\|\nabla \psi \right\|}^{2}={\left(\frac{\partial \psi }{\partial x}\right)}^{2}+{\left(\frac{\partial \psi }{\partial y}\right)}^{2}. & \;(13) \end{array}$$

To compare the CSD and LFP phase-patterns we used the *phase-coherence* between the simulated CSDs and LFPs, denoted by ρ_*LFP, CSD*_. Let ψ_*LFP*_ and ψ_*CSD*_ denote the LFP and CSD phase-patterns, respectively, where as mentioned above, the CSD has been restricted to the electrode locations. Then ρ_*LFP, CSD*_ is defined as

(14) $$\begin{array}{l} \rho_{LFP,CSD}=|\langle e^{i\left(\psi_{LFP}-\psi_{CSD}\right)}\rangle |, \end{array}$$

which takes values between 0 and 1 and quantifies the extent to which ψ_*LFP*_ and ψ_*CSD*_ are coherent.

## Results

Before systematically comparing LFP and CSD phase-patterns through simulations and identifying the factors that lead to discrepancies, in Section The LFP as an Integrated Signal we provide some intuition for the effects of volume-conduction and in Section Phase-Contraction we discuss a simple example that can be analyzed mathematically.

### The LFP as an integrated signal

Equation (2) is the formal solution of Poisson's equation and shows how the LFP arises from the volume density of transmembrane currents (the CSD): At any particular location, the LFP is generated by integrating the CSD over the tissue volume, where the CSDs contribution is weighted by the distance to the electrode tip.

Thus, in general, distant sources contribute less to the LFP than sources close to the electrode tip do. Equation (2) also shows that the effect of volume-conduction is *linear*: The LFP due to two sources is equal to the sum of the LFPs due to the individual sources. The LFP therefore is an integrated signal, reflecting the summed (or integrated) transmembrane currents from the surrounding tissue volume.

**Figure 3:**
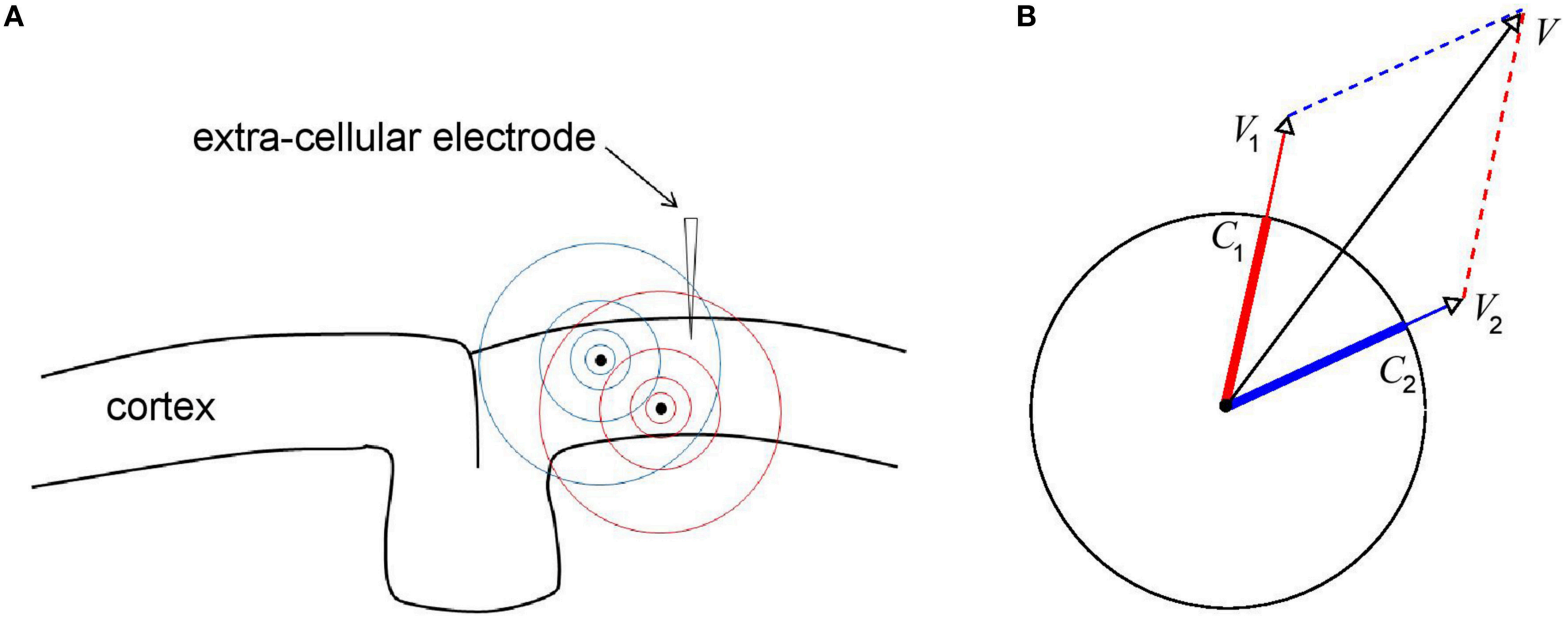
**Generation of LFPs through linear superposition. (A)** Shown is a piece of cortex with inserted an extra cellular recording electrode and two localized current sources (black dots). The electric fields generated by the sources are denoted by the blue and red concentric circles. The electric field, and hence the potential, at the recording tip, is equal to the linear superposition of the electric fields of the two individual sources. It is this (linear) integration property of the LFP that complicates its interpretation in terms of the dynamics of the underlying current sources. **(B)** Geometric interpretation of the effect of volume-conduction. Depicted are the instantaneous source variables (thick blue and red bars), the ensuing voltages *V*_1_ and *V*_2_ at the recorded tip (thin blue and red arrows), and the total voltage *V* at the recording tip (black arrow). All variables are represented by complex-valued numbers (that is, by an amplitude and a phase) and the figure illustrates how *V*_1_ and *V*_2_ at add up to produce *V*.

Figure 3A shows a piece of cortex with a recording electrode inserted into it and containing two current sources (black dots). The sources generate electric fields *E*_1_ and *E*_2_ (visualized in blue and red, respectively) with corresponding electric potentials *V*_1_ and *V*_2_ at the electrode tip (satisfying ∇*V*_1_ = −*E*_1_ and ∇*V*_2_ = −*E*_2_). The electric potential *V* due to both sources is given by the linear superposition of *V*_1_ and *V*_2_: *V* = *V*_1_+*V*_2_. It is this property of the LFP that complicates its interpretation (Buzsáki et al., 2012; Kajikawa and Schroeder, 2015) for it means that it reflects contributions from neural sources at different locations. In particular, LFPs recorded within a cortical layer, for example using the Utah array (Maynard et al., 1997), receive contributions from neural sources in all other cortical layers. Moreover, the amplitude of the LFP depends on the amplitudes *and* phases of all the neural sources and the same holds for the phase of the LFP. Thus, in contrast to the LFP itself, which depends linearly on the neural sources, its amplitude and phase depend on those of the neural sources in a non-linear way.

The geometric viewpoint illustrated in Figure 3B provides some intuition for how LFP amplitude and phase are related to those of the underlying CSD. The figure contains two oscillatory neural sources (*C*_1_ and *C*_2_) and the respective ensuing voltages (*V*_1_ and *V*_2_) represented by complex-valued numbers. Thus, at any given moment, they are characterized by an *amplitude* (length of the vectors) and a *phase* (angle of the vectors with the x-axis). Note that the sources have equal amplitudes and that the oscillations of *C*_2_ are delayed with respect to those of *C*_1_. Also note that the phases of *V*_1_ and *V*_2_ are equal to those of *C*_1_ and *C*_2_, respectively, reflecting the fact that the extra cellular space is purely resistive.

In Sections LFP-CSD Phase-Coherence, LFP and CSD Propagation Speeds, and Laminar Contributions to LFP oscillatory phase, we discuss differences between LFP and CSD phase-patterns. Specifically, in Section LFP-CSD Phase-Coherence we show that LFP phase-patterns are more spatially coherent than those of the underlying CSDs, in Section LFP and CSD Propagation Speeds we show that LFP traveling waves propagate faster than those of the underlying CSDs, and in Section Laminar Contributions to LFP oscillatory phase we show that the LFP phase is a weighted mixture of the phases of the CSDs from all cortical layers. Can we understand these effects from a geometric point of view? Figure 3B makes intuitively clear that the phase of *V* lies in between those of *C*_1_ and *C*_2_, which explains the effect discussed in Section Laminar Contributions to LFP oscillatory phase. This implies that when LFPs are recorded from two locations, the phase-difference between the LFPs is smaller than that between the underlying CSDs, an effect we will refer to as *phase-contraction* and prove it mathematically in the next section. The effects discussed in Sections LFP-CSD Phase-Coherence and LFP and CSD Propagation Speeds are direct consequences of this basic effect for it implies that LFP phases vary less over electrodes and hence are more spatially coherent (Section LFP-CSD Phase-Coherence) and that LFP phase-gradients are less steep, which implies faster propagation of traveling waves (Section LFP and CSD Propagation Speeds).

### Phase-contraction

In the special case of two monopolar sources, we can mathematically prove that volume-conduction leads to the contraction of CSD phases. Let the variables *C*_1_ = *a*_1_exp(*iφ*_1_) and *C*_2_ = *a*_2_exp(*iφ*_2_) denote the (complex-valued) CSDs of the monopolar sources, where *a*_1_ and *a*_2_ denote their amplitudes and φ_1_ and φ_2_ their phases and let us write ψ_*CSD*_ = φ_2_−φ_1_ for their phase-difference. Without loss of generality we assume that 0 < ψ < π and that *a*_1_, *a*_2_ > 0. The (complex-valued) LFP recorded at a given location is thus given by

(15) $$\begin{array}{l} V=\beta_{1}C_{1}+\beta_{2}C_{2}, \end{array}$$

for certain real-valued constants β_1_ and β_2_ that depend on the relative locations of the sources and electrode tips. Let us write φ_*LFP*_ for the LFP phase. We will show that φ_1_ < φ_*LFP*_ < φ_2_. Since the same argument can be applied to the LFP recorded from a second electrode, it follows that the phases of both LFPs lie in between φ_1_ and φ_2_. From the latter it follows that the LFP phase-difference ψ_*LFP*_ is smaller than the CSD phase-difference:

(16) $$\begin{array}{l} |\psi_{LFP}|<|\psi_{CSD}|, \end{array}$$

where the brackets denote taking the absolute value. The LFP phase-difference is thus *contracted*, relative to the CSD phase-difference.

To show that φ_1_ < φ_*LFP*_ < φ_2_, we first write *V* = ξβ_1_*C*_1_, where

(17) $$\begin{array}{l} \xi =1+\gamma \text{exp}\left(i\psi_{CSD}\right), \end{array}$$

with γ = β_2_*a*_2_∕β_1_*a*_1_ > 0. Note that φ_1_ < φ_*LFP*_ < φ_2_ is equivalent to 0 < ψ_ξ_ < ψ_*CSD*_, where ψ_ξ_ denotes the phase of ξ.

To show that ψ_ξ_ < ψ_*CSD*_, note that

$$\begin{array}{ll} \psi_{\xi }<\text{arctan}\left(\frac{\gamma \text{sin}\left(\psi_{CSD}\right)}{1+\gamma \text{cos}\left(\psi_{CSD}\right)}\right)\\ \; & \;<\text{arctan}\left(\frac{\text{sin}\left(\psi_{CSD}\right)}{\text{cos}\left(\psi_{CSD}\right)}\right)=\psi_{CSD}. \end{array}$$

From the above expression of ψ_ξ_ in terms of arctan it also follows that ψ_ξ_ > 0, which completes the argument.

### LFP-CSD phase-coherence

LFP phase-dynamics exhibit a range of dynamical patterns, including plane and spiral waves and local contractions and expansions (Townsend et al., 2015). Moreover, phase-gradients are typically coherent only over short distances and propagation velocities (both magnitude and direction) can vary between nearby locations (Rubino et al., 2006; Kral et al., 2009). In addition, complex wave patterns are also frequently observed using voltage sensitive dye (VSD) imaging of cortical tissue (Wu et al., 2008), which is not affected by volume-conduction and therefore further supports the existence of such patterns on the level of neural activity itself (in the sense of membrane depolarization). Given these and related observations, it is relevant to map the discrepancies between complex CSD and LFP phase-patterns.

**Figure 4:**
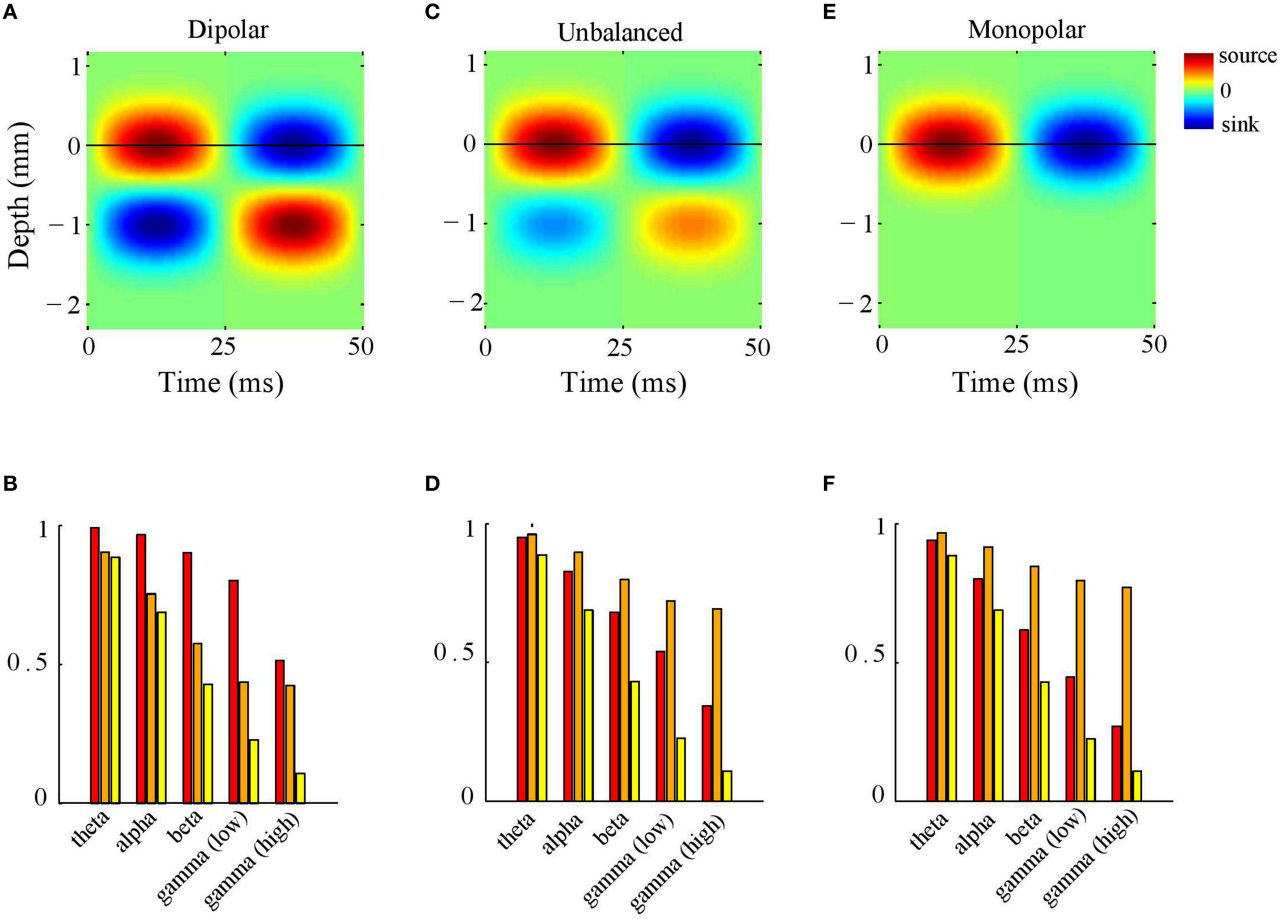
**LFP-CSD phase-coherence. (A)** Simulated inter-laminar oscillatory CSD profile as a function of time and cortical depth (relative to the electrode grid). The CSD values are color coded: red and blue correspond to sources (CSD > 0) and sinks (CSD < 0), respectively. The CSD profile is modeled by a generator of length 1 mm, located at a depth of 0.5 mm. For this illustration, the temporal frequency was set to 20 Hz. The black horizontal line designates the electrode grid. **(B)** LFP–CSD spatial phase-coherence (red bars) and LFP (orange bars) and CSD (yellow bars) Kuramoto order parameters for each of the five frequency bands (theta, alpha, beta, low and high gamma). Results were obtained by averaging over 500 realizations. **(C,D)** Same as **(A,B)** but with the amplitude of the deep generator pole set to half its value. **(E,F)** Same as **(A,B)** but with the amplitude of the deep generator pole set to zero.

To this end, we simulated CSD phase-patterns by superimposing 100 isotropically propagating waves with random intra-laminar locations and random initial phases (see Section CSD Simulations). The inter-laminar CSD profile was modeled by a generator with length 1 mm and located at a depth of 0.5 mm (relative to the electrode grid) as shown in Figure 4A. Figure 4B shows the LFP-CSD phase-coherence (red bars) and LFP and CSD Kuramoto order parameters (orange and yellow bars, respectively) for each of the five frequency bands. The values were obtained by averaging over 500 realizations. Note that for the lower frequency bands (theta, alpha, and beta) the LFP-CSD phase-coherence is close to one but decreases markedly for gamma band oscillations. Thus, while for low-frequency oscillations, LFP phases accurately reflect CSD phases at the corresponding locations, for high-frequency oscillations they become increasingly inaccurate. This can be understood by looking at the values of the Kuramoto order parameter. Specifically, while the CSD Kuramoto order parameter steadily decreases with increasing frequency (yellow bars), the LFP Kuramoto order parameter decreases slower and converges to a non-zero value (orange bars). This means that for high-frequency oscillations (low and high gamma), the LFP phases are spatially more coherent than the CSD phases and in fact reach a minimum level of spatial coherence. As a result, the discrepancy between LFP and CSD phases decreases with increasing frequency.

**Figure 5:**
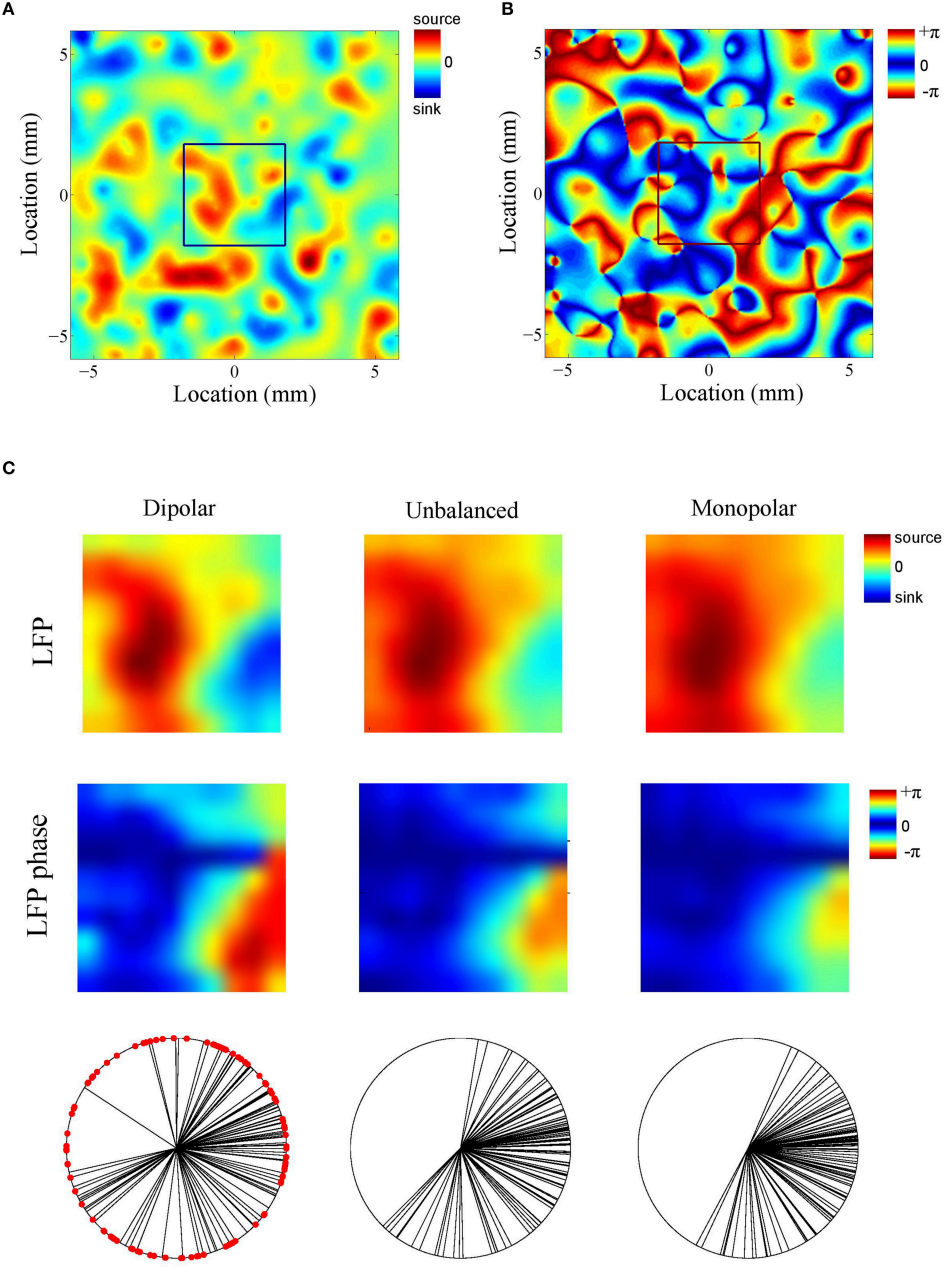
**Example (beta oscillations). (A,B)** Single realization of the simulated intra-laminar CSD profile **(A)** and its phase **(B)** in the case of beta (20 Hz) oscillations. The black square in the center of the figures designates the boundary of the electrode grid. **(C)** Corresponding LFP (top row) and its phase (middle row) for the (balanced) dipolar CSD (first column), the unbalanced CSD (middle column), and the monopolar CSD (right column). The bottom row contains circular plots of the LFP phases. Each black line corresponds to an electrode. The red dots in the left panel correspond to the CSD phases at the electrode locations (which remain the same for the three simulations).

What is the role of the deep generator pole? Given that the electric potential of a monopolar source is inversely proportional to distance, one might expect its contribution to be modest. To assess this, we repeated the above simulation with the difference that the amplitude of the deep generator pole was set to half its value (see Figure 4C). The results are shown in Figure 4D. The figure shows that the LFP-CSD phase-coherence now drops much faster with increasing oscillation frequency (red bars) and that the LFP Kuramoto order parameters converges to a higher value than in Figure 4B.Thus, an imbalance in the inter-laminar CSD profile causes the LFP phases to become more spatially coherent, which leads to a larger discrepancy with the CSD phases for fast oscillations (beta and gamma). We also repeated the simulations while setting the amplitude of the deep generator pole to zero (see Figure 4E). Figure 4F shows that, as expected, the discrepancy between LFP and CSD phases further increases. The difference between the unbalanced (Figure 4D) and the monopolar (Figure 4F) simulations, however, is much smaller than between the unbalanced and balanced (Figure 4B) simulations. Figure 5 shows a single realization in the case of beta band oscillations. It shows that imbalancing the inter-laminar CSD component leads to increased LFP spatial coherence by “contracting” the LFP phases (see bottom row).

### LFP and CSD propagation speeds

In this section we asses differences between LFP and CSD propagation velocities in case when the intra-laminar CSD profile is modeled as a traveling plane wave (Section CSD Simulations). Because LFP and CSD propagation *directions* are the same, at least for electrically isotropic tissue, we only consider their *speeds*, that is, the magnitudes of their velocity vectors.

**Figure 6:**
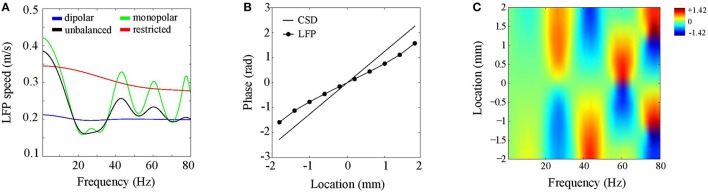
**Discrepancies between LFP and CSD propagation speeds. (A)** LFP propagation speed as a function of frequency. The unbalanced (black line) and monopolar (blue line) cases correspond to setting the amplitude of the (dipolar) generator to half its value and to zero, respectively. The red line corresponds to the dipolar case but with restricting the intra-laminar CSD profile to the electrode grid. **(B)** CSD (solid line) and LFP (circle markers) phase as a function of intra-laminar location for 40 Hz oscillations. The phases were obtained from a cross-section through the electrode grid perpendicular to the propagation direction. **(C)** Color-coded difference between the LFP and CSD phases as a function of intra-laminar location and oscillation frequency. Color-scale is in radians.

We consider CSDs with an inter-laminar profile comprising a single generator of 1 mm and located at a depth of 0.5 mm (see Figure 4A). Such a generator models synaptic activation of deep pyramidal neurons, which have extended apical dendrites that give rise to an elongated dipolar sink/source configuration. As described in Section CSD Simulations, the CSD propagation speed was 0.2 m/s. Figure 6A shows LFP propagation speed as a function of frequency (blue line). Notice that over the entire frequency range, the LFP speed is close to 0.2 m/s. Small perturbations of the generator depth or width of its poles left the LFP speed practically unchanged. Furthermore, the LFP speed remained unchanged when the initial phase of the CSD waves was varied (results not shown). Thus, in case of balanced (dipolar) inter-laminar CSD profiles, LFP propagation speeds truthfully reflect those of the underlying transmembrane currents.

To assess the effect of imbalancing the generator, we proceed similarly as in the previous section, namely, by repeating the above simulation with the difference that the amplitude of the deep generator pole is set to half its value (black line in Figure 6A) or to zero (green line in Figure 6A). Note that the LFP speed depends on the oscillation frequency and can either be lower or higher than the CSD speed. This complex dependency on oscillation frequency reflects the non-linear effect of volume-conduction on the phases of (oscillatory) neural currents. This simulation shows that balanced transmembrane currents are responsible for the correspondence between LFP and CSD propagation speeds (blue line). The CSD oscillations in deep cortical layers, which are in anti-phase with those at the electrode plane, reduce the (mostly intra-cortical) effect of volume-conduction. Imbalances in transmembrane currents do exist (Riera et al., 2012) and our simulations could explain why experimentally observed propagation speeds of cortical LFPs are confined to the range of 0.1–0.3 m/s (Freeman et al., 2000; Rubino et al., 2006; Reimer et al., 2011; Takahashi et al., 2011; Zanos et al., 2015), like they do in Figure 6A. Another factor that might underlie variations in experimentally observed LFP speeds is the intra-laminar source extent. This is suggested by the red line in Figure 6A, which shows that LFP speed markedly increases when the intra-laminar CSD profile is confined to the electrode grid.

Another non-linear feature of the LFP waves is that, in contrast to the CSD waves, their speed depends on location. Figure 6B displays the CSD and LFP phases as a function of location in the case of 40 Hz oscillations. These were obtained by taking a cross-section of the electrode grid in the direction perpendicular to the traveling direction of the waves. The figure shows that the LFP phase does not linearly increase with location. In particular, in the middle of the grid (0 mm) the LFP wave is faster than the CSD wave while at the grid edges they are roughly equal. Figure 6C shows that this spatial-dependence of LFP propagation speed varied with LFP oscillation frequency. In fact, while LFP speed is constant for low frequencies, for which CSD wavelengths are much larger than the electrode grid, spatial-dependencies arise for frequencies for which CSD wavelengths are of the same order of magnitude as the electrode grid. There are hence three regimes for the CSD wavelength λ that are relevant for the behavior of the LFP phase-gradient: much larger, of similar size, and much smaller than the electrode grid. In the large−λ limit, LFP propagation speed is constant (and equals about 4.2 m/s), in the small−λ limit, LFP propagation speed diverges, eventually leading to LFP standing waves, while in between, volume-conduction gives rise to complex spatial-dependencies in LFP speed.

### Laminar contributions to LFP oscillatory phase

The simulations in Sections LFP-CSD Phase-Coherence and LFP and CSD Propagation Speeds assumed neural currents whose inter-laminar profile comprised a single generator. Cortical oscillations, however, arise via synaptic feedback between neural populations in different layers and one might therefore expect to observe inter-laminar phase-differences (Bastos et al., 2012). Several kinds of cortical as well as hippocampal oscillations indeed show inter-laminar phase-differences (Schroeder et al., 1998; Bollimunta et al., 2008; Lubenov and Siapas, 2009; Csercsa et al., 2010; Fourcaud-Trocmé et al., 2014). A relevant question, therefore, is how and to what extent these phase-differences shape the oscillatory phase of the LFP, recorded at a particular depth. Indeed, because neural currents across cortical layers contribute to the generation of the LFP, how are we to interpret its phase in the presence of inter-laminar phase-differences? The answer to this question is particularly relevant for the interpretation of spike-LFP coherences, spike-triggered LFPs, and for phase-based coding and long-range synchronization in general (Fries, 2005; Maris et al., 2016).

**Figure 7:**
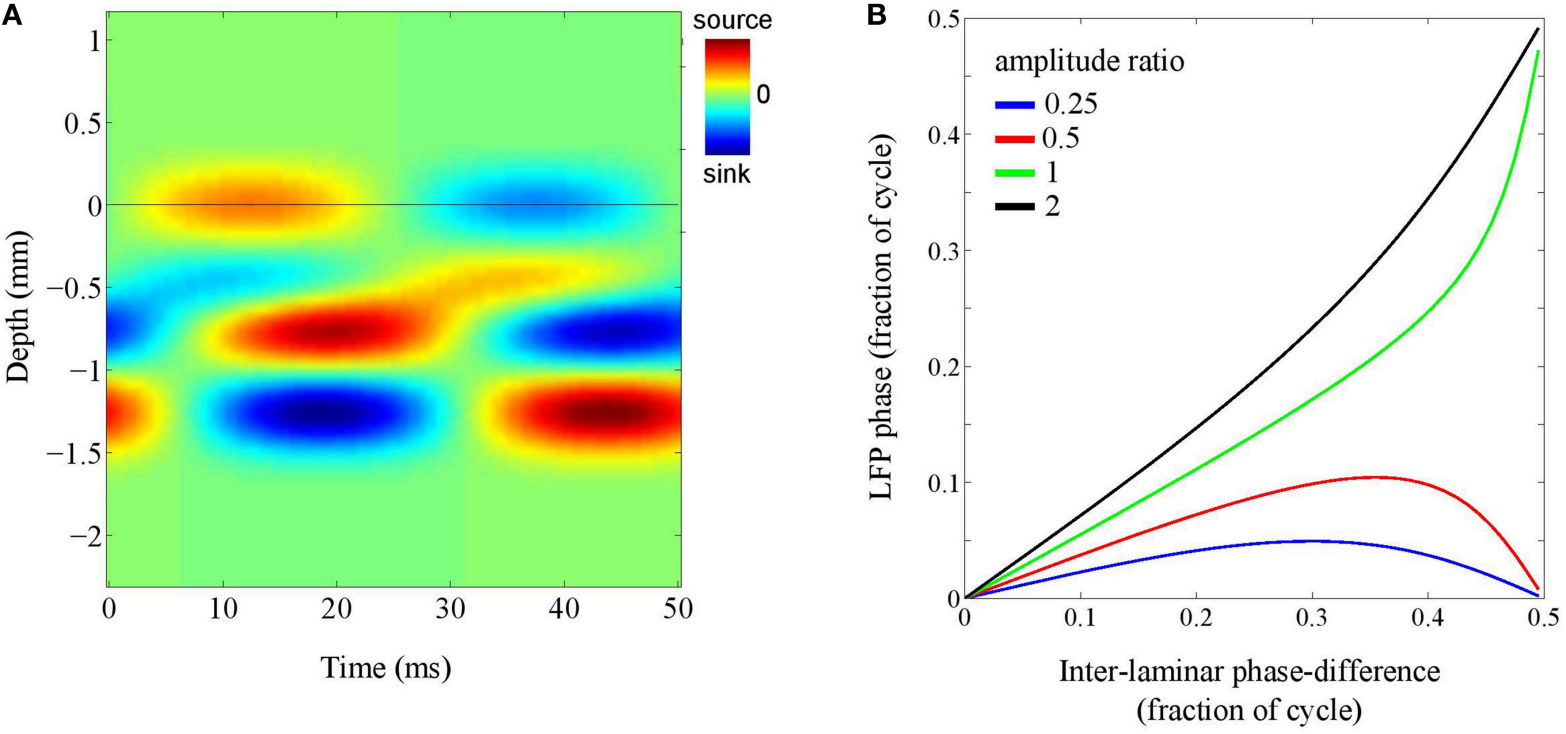
**Laminar contributions to LFP phase. (A)** Simulated inter-laminar CSD component as a function of time and cortical depth (relative to the electrode grid). The CSD values are color coded: red and blue correspond to sources (CSD > 0) and sinks (CSD < 0), respectively. The CSD profile is modeled by a superposition of two generators with common length 1 mm and located at depths of 0.25 and 1 mm. For this illustration, the temporal frequency was set to 20 Hz. The black horizontal line designates the electrode grid. In this example, the phase-difference between the oscillations in the superficial and deep layers is one-eighth of the oscillation period (π∕4) and the amplitude of the deep generator is twice that of the superficial. The black horizontal line denotes the electrode grid. **(B)** LFP phase as a function of the phase of the deep generator for four different amplitude ratio's (deep over superficial): 1/4 (blue), 1/2 (red), 1 (green), and 2 (black).

To address this question, we simulated a localized source whose inter-laminar profile was modeled by the superposition of two generators: a superficial and a deep generator, located at depths of 0.25 mm and 1.00 mm, respectively, and having a common length of 0.5 mm. The phase-difference between the oscillations in the deep and superficial layers was allowed to vary (see Figure 7A). The intra-laminar CSD profile was modeled as an isotropically propagation wave with a spatial width of 1 mm and setting the propagation speed to infinity, thereby effectively simulating a source with a Gaussian profile. The recording electrode was placed at the intra-laminar center of the source. The phase of the superficial generator was set to zero and the phase of the deep generator was varied from zero to half the oscillation cycle (π rad).

Figure 7B shows the results for four different amplitude ratio's of the deep generator over the superficial generator (1/4, 1/2, 1, and 2). It shows, for each of the four amplitude ratios, the LFP phase as a function of the phase of the deep generator. Note that in the absence of the deep generator, the curve would be horizontal (LFP phase equal to zero). On the other hand, in the absence of the superficial generator, the curve would be diagonal (LFP phase equal to deep generator phase). As a consequence, with increasing amplitude of the deep generator, the curve moves from being horizontal to being diagonal, but necessarily ends at either 0 or 0.5 (note that in the figure, phases are measured in fractions of the oscillation cycle). The latter can be understood by taking a geometrical perspective (Section The LFP as an Integrated Signal): If two sources are out-of-phase, they lie on a straight line in the complex plane and therefore, any linear combination lies on this line as well. In other words, any pair of LFPs that is due to a pair of out-of-phase CSDs is either in- or out-of-phase. In particular, if the ratio between the generator amplitudes is increased from 1/2 to 1, the end of the curve jumps from 0 to 0.5. This ensures that the LFP phase remains close to that of either the deep or the superficial generator, say within one-tenth of the oscillation cycle.

Because the phase-shifts, as measured relative to the oscillation cycle, are independent of the oscillation frequency, this result suggests that generally, and in the absence of knowledge about the laminar organization and phase-differences of the current generators, the LFP phase cannot be assumed to follow the phase of the underlying current oscillations with millisecond precision. Furthermore, the phases at which physiological or behavioral events are locked to the LFP are exact only up to a fraction of the oscillation cycle. For example, systematic phase-differences between LFP and voltage-sensitive dye (VSD) oscillations can be observed (Lippert et al., 2007), which can be understood by our simulations. In particular, while VSD signals predominantly reflect neural activity from superficial cortical layers, LFPs contain contributions from all layers, which explains the generally low correlation between LFP and VSD signals (Lippert et al., 2007) and systematic phase-differences in particular.

### The average-reference montage

Electrical potentials are relative measurements in that they are measured with respect to the potential at another electrode. In our simulations so far, we have considered “absolute” potentials, that is, recorded with respect to infinity. In practice, however, the reference electrode has to make contact with the volume conductor and is often placed on cortical tissue that is far from the electrode grid (for example on the other hemisphere) or on a piece of bone. This measurement configuration or *montage* is referred to as the *referential (or unipolar) montage* and is the most used montage in two-dimensional LFP recordings (Menzel and Barth, 2005; Rubino et al., 2006; Lubenov and Siapas, 2009; Takahashi et al., 2011; McDonald et al., 2014; Zanos et al., 2015). A reference electrode is never “quit” however and as a consequence, the voltage fluctuations measured at the active electrodes are contaminated and can become difficult to interpret. Another difficulty in interpreting referential LFPs is the possible contribution of distant sources, which are not necessarily located directly underneath the electrode grid (Kajikawa and Schroeder, 2011). Because the distance between the reference electrode is typically much larger than the mutual distances between the active electrodes, all active electrodes are contaminated by *the same* fluctuations and the same holds (approximately) for distant sources. Therefore, these contaminations can be removed by subtracting the electrode-averaged voltage from the voltage measured at each electrode, a procedure referred to as re-referencing to the *average-reference montage*.

The average-reference montage is frequently applied to scalp EEG and ECoG recordings and less to LFP recordings (but see Hall et al., 2014). For scalp EEG recordings, it is motivated by the fact that for balanced current sources inside a closed volume conductor, the integral of the electric potential over the boundary of the conductor equals zero. Therefore, since the head can be treated (to a certain extent) as a closed volume conductor, average referenced EEG potentials are approximately equal to the absolute potentials, provided that a sufficient part of the head is covered by electrodes (Nunez et al., 1997). It is not clear however, to what extent the average-reference montage can be applied to multi-electrode LFP recordings without distorting them and rendering them uninterpretable. This is a relevant issue because if justified, the average-reference montage allows removing the contributions from the reference electrode as well as from distant sources.

**Figure 8:**
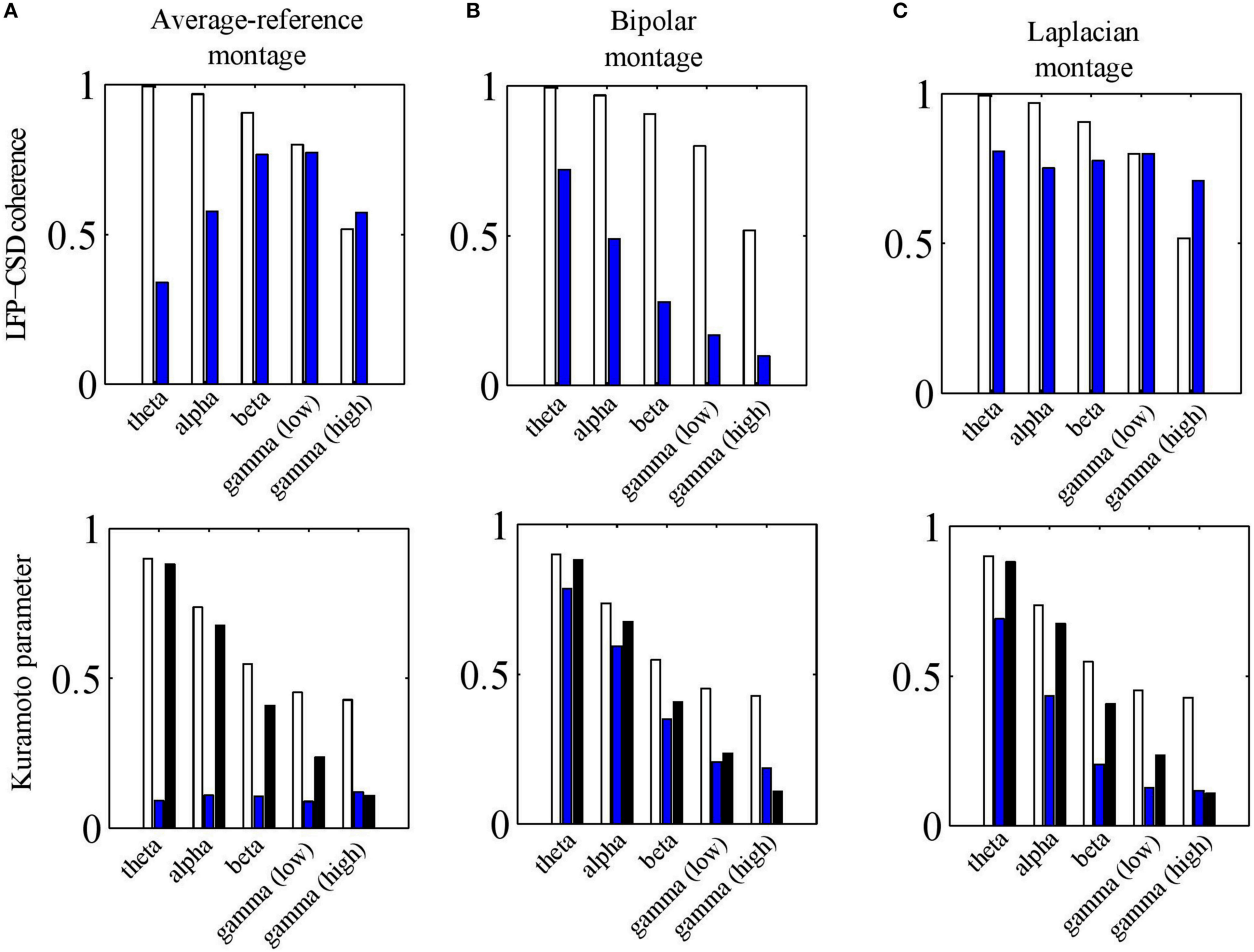
**Effects of electrode montages. (A)** LFP-CSD phase-coherence (upper panel) and Kuramoto order parameter (lower panel) for the average-reference montage (blue bars) for each of the five frequency bands (theta, alpha, beta, low and high gamma). The LFP-CSD phase-coherences (upper panel) and Kuramoto order parameters (lower panel) for the referential montage (white bars) are shown for comparison. The black bars in the lower panel denote the CSD Kuramoto order parameters. **(B**,**C)** Same as **(A)** but for the bipolar montage **(B)** and Laplacian montage **(C)** instead of the average-reference montage **(A)**. In **(B)** the results are shown for the bipolar montage in the *x*− direction only. The values were obtained by averaging over 500 simulations.

To address this question, we repeated the simulations of Section LFP-CSD Phase-Coherence and calculated the LFP-CSD phase-coherence and the CSD and LFP Kuramoto order parameters for both the absolute and the average-reference montages. The results are summarized in Figure 8A. The lower panel shows that passing to the average-reference montage removes the spatially-coherent component of the LFP (blue bars). When this LFP component only contains volume-conducted activity from distant sources or fluctuations at the reference electrode, removing this component can be beneficial. This can be seen in the high gamma band, in which the simulated oscillations are spatially incoherent so that switching to the average-reference montage (slightly) increases the LFP-CSD phase-coherence (top panel). In the simulations it are not distant sources or fluctuations at the reference electrode that are removed, but the contribution of neural currents in cortical layers other than in which the electrode grid is located. In any case, in the higher frequency bands—in which the neural oscillations are relatively spatially incoherent—switching to the average-reference montage does not lead to a higher discrepancy between CSD and LFP phases. In the lower frequency bands (up to the beta band) the simulated oscillations are spatially more coherent and therefore, switching to the average-reference montage removes part of the source activity. This leads to a lower LFP-CSD phase-coherence and hence makes the LFP phases more difficult to interpret.

### The bipolar montage

The *bipolar montage* is also frequently used in EEG and ECoG studies and is obtained by taking the differences between neighboring electrodes (in a given direction). Like the average-reference montage, the bipolar montage suppresses contributions from the reference electrode and from distant sources. Its physical interpretation, however, is different. The spatial derivative of *V* in a direction *a* is given by

$$\begin{array}{l} \frac{\partial V}{\partial a}=\nabla V•a=-E•a, \end{array}$$

where ∇ denotes the gradient operator and *E* the extra cellular electric field. Note that ∂*V*∕∂*a* corresponds to the bipolar montage in the direction of *a*. Furthermore, since nervous tissue is purely resistive, the extra cellular current density *J* is related to *E* by *J* = σ*E*. Therefore, the bipolar montage is proportional to the current density in that direction. The current density, however, is not equal to the CSD (they are related through ∇•*J* = *C*) so that the bipolar montage is expected to lead to a larger discrepancy between LFP and CSD phases. As Figure 8B shows, this is indeed the case: passing to a bipolar montage substantially reduces LFP-CSD phase-coherence in all frequency bands and thereby making LFP phases more difficult to interpret.

### The laplacian montage

A montage that is frequently used in one-dimensional (laminar) LFP recordings is the *Laplacian montage* (Mitsdorf, 1985). It is directly motivated by Poisson's equation (Equation 1). In Cartesian coordinates (*x, y, z*), where (*x, y*) and *z* denote intra- and inter-laminar locations, respectively, Poisson's equation is given by

(18) $$\begin{array}{l} \left(\sigma_{x}\frac{\partial^{2}}{\partial x^{2}}+\sigma_{y}\frac{\partial^{2}}{\partial y^{2}}+\sigma_{z}\frac{\partial^{2}}{\partial z^{2}}\right)V\left(x,y,z\right)\;=\;-C\left(x,y,z\right), \end{array}$$

where we have assumed that the conductivity tensor σ is diagonal with entries σ_*x*_, σ_*y*_, and σ_*z*_. It follows that if *C* is constant in the intra-laminar directions, Poisson's equation reduces to

(19) $$\begin{array}{l} \frac{\partial^{2}V}{\partial z^{2}}\left(z\right)=-\frac{1}{\sigma_{z}}C\left(z\right), \end{array}$$

so that the second-order spatial derivative of *V* in the inter-laminar direction can be used to “invert” Poisson's equation and estimate the inter-laminar component of the CSD. For this reason, the Laplacian montage is also referred to as the *CSD method* (Nicholson and Freeman, 1975). Note that the CSD method assumes σ_*z*_ to be constant across cortical layers, an assumption that is questionable (Goto et al., 2010).

The Laplacian montage can also be used in two dimensions as is regularly done in scalp EEG recordings (Nunez et al., 1997; Tenke and Kayser, 2012). In the context of scalp EEG recordings, the two-dimensional Laplacian montage is referred to as the *surface Laplacian* and reflects the currents that entering and leaving the scalp (scalp sources and sinks). Under some extra assumptions, the surface Laplacian can be shown to be approximately equal to the dura potential so that is can be used to “invert” the blurring effect of the skull and scalp on the electric potential (Nunez et al., 1997).

In the case of (intra-cortical) LFP recordings, the surface Laplacian is directly related to neural membrane currents. Specifically, if *C* is constant in the inter-laminar direction and assuming that cortical conductivity is independent of intra-laminar direction (say $\sigma_{x}=\sigma_{y}=\sigma^{\prime }$ for certain σ′) then

$$\begin{array}{l} \left(\frac{\partial^{2}}{\partial x^{2}}+\frac{\partial^{2}}{\partial y^{2}}\right)V\left(x,y\right)=-\frac{1}{\sigma^{\prime }}C\left(x,y\right). \end{array}$$

The (surface) Laplacian montage can hence be used to estimate the intra-laminar CSD component *C*(*x, y*). Note that in Section CSD Simulations, we have used the notation *C*_*v*_ and *C*_*h*_ for the inter- and intra-laminar components of *C*, respectively. While the conductivity assumption seems generally satisfied (Logothetis et al., 2007), the assumption on *C* is clearly not since the cortical sheet has finite thickness and, perhaps more importantly, the inter-laminar CSD profile is often balanced, and in any case, is not constant. Although the surface Laplacian montage is not commonly used in the analysis of multi-electrode LFP recordings, it is certainly interesting, as it potentially allows recovering the source distribution in the electrode plane. It will therefore be relevant to see if it can still be recovered even if one of the assumptions is violated.

To assess this issue, we carried out the same simulations as for the other electrode montages (see Sections The Average-Reference Montage and The Bipolar Montage) and calculated the LFP-CSD phase-coherence and LFP and CSD Kuramoto order parameters. Note that in these simulations, the assumption of a constant inter-laminar CSD profile is violated since the profile is balanced. The results are summarized in Figure 8C.The upper panel shows that the LFP-CSD phase-coherences are rather independent of frequency and are relatively high (around 0.75) indicating a reasonable correspondence between (Laplacian-referenced) LFP and CSD phases. The effect of the finite thickness of the cortical sheet is modest as we verified by simulating CSDs with a constant inter-laminar profile: the resulting CSD-LFP phase-coherences were mostly > 0.9 for all frequency bands (results not shown).

## Discussion

The phases of oscillatory LFPs have been tied to cognitive, perceptual, and motor processing and their organization in space is thought to implement a basic mechanism of neural processing (Fries, 2005; Deco and Kringelbach, 2016; Maris et al., 2016). To further advance our understanding of how neural oscillations subserve cognition, it is crucial to understand how LFP phases are related to those of the underlying neural activity. LFPs are generated by the extra cellular electric fields that are induced by neural activity, that is, by transmembrane currents (Buzsáki et al., 2012). Although transmembrane currents and extra cellular potentials are different physical quantities, in experimental studies, LFPs are often equated with neural activity. In this study we made this assumption explicit and used a volume-conductor model to investigate the relation between the phases of oscillatory transmembrane currents—as modeled by current source densities (CSDs)—and those of the ensuing LFPs. Although we have focused on two-dimensional LFP recordings using Utah arrays (Maynard et al., 1997), the results are relevant to other electrode configurations as well. Importantly, we found that discrepancies between LFP and CSD phase-patterns do exist and can be substantial. One finding is that, in the case of inter-laminar phase-differences, LFP phases cannot be associated with those of the CSD at any particular cortical depth, but rather, are shaped by the phases and amplitudes of the CSD oscillations in all cortical layers. This can explain the low correlations and systematic phase-differences between VSD and LFP signals as suggested earlier in Lippert et al. (2007). The two other main findings are the following. First, the most important factors that determine the discrepancy between LFP and CSD phases are the frequency of the cortical oscillations and the extent to which their laminar CSD profile is balanced. Second, although not commonly used in two-dimensional LFP recordings, switching to the Laplacian montage increases the correspondence between CSD and LFP phases and hence renders the LFP phase easier to interpret. This is in contrast to the average-reference montage, which leads to larger LFP-CSD discrepancies, particularly for low-frequency oscillations, and renders LFP phases difficult to interpret. It will certainly be interesting to see how, and to what extent, the relation between LFP phase-dynamics and behavioral indices, for example those reported in Riehle et al. (2013), Hall et al. (2014), Best et al. (2016), changes when switching to the Laplacian montage.

As mentioned above, the two main factors that determine the discrepancy between LFP and CSD phases are the frequency of the cortical oscillations and the extent to which their laminar CSD profile is balanced. With respect to their oscillation frequency, we note that this is important only indirectly. What effectively matters is the (intra-laminar) spatial frequency spectrum of the oscillations. Since the wavelength of the simulated isotropic sources is given by λ = *v*∕*f*, where *v* is their propagation speed and *f* their (temporal) frequency, a higher frequency leads to shorter wavelengths, that is, to higher spatial frequencies. Besides the oscillation frequency, however, there are other factors that shape the oscillations' spatial frequency spectrum. These are the amplitudes, initial phases, and number of isotropic sources. Thus, the (temporal) frequency is just one of several factors that shape the oscillations' spatial frequency spectrum and thereby determine the correspondence (or lack thereof) between LFP and CSD phases. With respect to the inter-laminar CSD profile, our main finding is that LFP recordings of unbalanced sources are more contaminated by volume-conduction. Perhaps surprisingly, halving the amplitude of the deep generator's pole decreases the correspondence between LFP and CSD phases almost as much as in the case of a monopolar source. In other words, discrepancies between LFP and CSD phases do not arise *because of* but *in spite of* the deep generator poles and are hence mostly due to volume-conduction in the intra-laminar directions.

Our findings allow an interpretation for the propagation speeds of cortical LFP traveling waves, which typically fall within the range 0.1–0.3 m/s (Freeman et al., 2000; Rubino et al., 2006; Lubenov and Siapas, 2009; Reimer et al., 2011; Zheng and Yao, 2012; Patel et al., 2013; Zanos et al., 2015), and provide an explanation for the variation within this range. Based on our simulations, there are two possible interpretations. First, if LFP recordings are dominated by currents in deep cortical layers (relative to the electrode grid), LFP propagation speed is about an order of magnitude higher than CSD propagation speed (results not shown). This would imply that the CSD propagation speeds are around 0.02 m/s. Although such low propagation speeds have been observed using VSD imaging (Kleinfeld et al., 1994; Wu et al., 2008; Sato et al., 2012), they mostly pertain to low-frequency activity, while the range 0.1–0.3 m/s is observed across the frequency spectrum. Moreover, propagation speeds of VSD traveling waves usually fall within this last range (Slovin et al., 2002; Benucci et al., 2007; Wu et al., 2008; Sato et al., 2012). The more likely scenario, therefore, is that the recorded LFPs are largely generated by current sources close to the electrode tips. Our simulations have shown that in this scenario, LFP speeds are only moderately higher than CSD speeds. Of interest to note is that one-dimensional LFP recordings in human hippocampus have estimated the propagation speeds of theta oscillations to be 2–5 m/s (Zhang and Jacobs, 2015), that is, an order of magnitude higher than those reported by Utah array recordings. Based on our simulations, likely explanation is that the electrode tips are located further away from the neural generators, leading to large increases in propagation speeds. The same probably holds for ECoG and scalp EEG oscillations, whose speeds fall in the same range (Bahramisharif et al., 2013; Hindriks et al., 2014). Concerning variation in propagation speed across experiments, our simulations have shown that when CSD oscillations propagate with 0.2 m/s, the ensuing LFP oscillations propagate with speeds in the range 0.2–0.4 m/s, depending on the laminar profile of the oscillations, their frequency, and their (intra-laminar) spatial extent.

LFPs are recorded using a distant reference electrode and can subsequently be converted to a different montage if desired. In two-dimensional LFP recordings, the data are usually not re-referenced during their analysis (Menzel and Barth, 2005; Rubino et al., 2006; Lubenov and Siapas, 2009; Takahashi et al., 2011; McDonald et al., 2014; Zanos et al., 2015). Apart from electrical activity at the reference electrode, the presence of distant sources, and possible muscle artifacts, all of which can contaminate the recorded signals, our simulations have shown that using this montage, the discrepancy between LFP and CSD phases increases with increasing oscillation frequency. Specifically, for high-frequency oscillations, the LFP phases are more spatially coherent than the CSD phases. Switching to the average-reference montage, as done in some studies (Hall et al., 2014), increases the discrepancy between LFP and CSD phases because this montage completely removes the spatially coherent component in the data. Thus, the average-reference is only useful when the neural activity is known to be spatially incoherent, at least down to the scale of the inter-electrode distances (0.4 mm in our simulations). Switching to a bipolar montage makes things even worse. The (two-dimensional) Laplacian montage, however, was found to produce only moderate discrepancies between LFP and CSD phases throughout the entire frequency range (1–80 Hz). We mention that in this study we only carried out a theoretical evaluation of the Laplacian montage. In practice, the presence of measurement noise requires certain interpolation schemes to be used, which might lead to suboptimal results (Nunez et al., 1997; Tenke and Kayser, 2012). This, however, is a practical question that falls outside the scope of this study.

Switching to the Laplacian montage can be viewed of as “inverting” Poisson's equation that links the LFP to the CSD and this is in fact what motivates this montage. A more general way of “inverting” Poisson's equation, which allows to incorporate the geometry and electrical properties of the tissue as well as any *a priori* knowledge of the current sources, is to construct a forward model and to subsequently invert it, a technique that is widely applied within the field of EEG and MEG (Grech et al., 2008). In contrast, inverse modeling of field potentials, either ECoG or LFPs, is only now becoming more common (Pettersen et al., 2006; Zhang et al., 2008; Leski et al., 2011). The discrepancies between LFP and CSD phases uncovered by our simulations further motivate the use of such inverse methods in the analysis and interpretation of experimental LFPs.

Our findings are also relevant for computational modeling studies of LFPs. Several studies have been devoted to modeling the physiological mechanisms underlying LFP (and VSD) traveling waves and have done so on different levels, ranging from spiking neurons endowed with a multitude of intrinsic currents to neural fields (Sanchez-vives and Mccormick, 2000; Ermentrout and Kleinfeld, 2001; Compte et al., 2003; Kumar et al., 2008; Destexhe, 2009; Coombes, 2010; Heitmann et al., 2012). In such studies, LFPs are commonly assumed to be proportional to membrane voltage or synaptic currents and the geometric, and in particular, the inter-laminar organization of the modeled neural activity is often given less attention, although this considerably impacts the LFP (Buzsáki et al., 2012; Reimann et al., 2013). For example, LFP features depend on the distribution of receptors along the apical dendrites of deep pyramidal cells, because each distribution sets up a specific three-dimensional CSD density. Our study demonstrates the importance of using forward models in computational studies on LFPs. Complementing computational models with suitable forward models might improve the match between simulated and experimental observations and enable more concrete predictions to be formulated.

## Author contributions

RH and XA, TP, and MB designed the study, RH and XA wrote the code and conducted the simulations, RH wrote the paper, and PV, NL, and GD supervised the study.

### Conflict of interest statement

The authors declare that the research was conducted in the absence of any commercial or financial relationships that could be construed as a potential conflict of interest.
